# Study on the Time-Dependent Mechanical Behavior and Springback of Magnesium Alloy Sheet (AZ31B) in Warm Conditions

**DOI:** 10.3390/ma14143856

**Published:** 2021-07-09

**Authors:** Jae-Hyeong YU, Chang-Whan Lee

**Affiliations:** Department of Mechanical Design and Manufacturing Engineering, Seoul National University of Science and Technology, Seoul 01811, Korea; jhyu9109@seoultech.ac.kr

**Keywords:** time-dependent springback, creep, stress-relaxation, AZ31B, magnesium alloy

## Abstract

In this study, the time-dependent mechanical behavior of the magnesium alloy sheet (AZ31B) was investigated through the creep and stress relaxation tests with respect to the temperature and pre-strain. The microstructure changes during creep and stress relaxation were investigated. As the tensile deformation increased in the material, twinning and dynamic recrystallization occurred, especially after the plastic instability. As a result, AZ31B showed lower resistance to creep and stress relaxation due to dynamic recrystallization. Additionally, time-dependent springback characteristics in the V- and L-bending processes concerning the holding time and different forming conditions were investigated. We analyzed changes of microstructure at each forming temperature and process. The uniaxial tensile creep test was conducted to compare the microstructures in various pre-strain conditions with those at the secondary creep stage. For the bending process, the change of the microstructure after the forming was compared to that with punch holding maintained for 1000 s after forming. Due to recrystallization, with the holding time in the die set of 60 s, the springback angle decreased by nearly 70%. Increased holding time in the die set resulted in a reduced springback angle.

## 1. Introduction

Magnesium alloy is an HCP (hexagonal closed packed) structure. It has fewer slip planes at room temperature than BCC (body-centered cubic) or FCC (face-centered cubic) structures [1]. Due to its structure, magnesium alloy has a higher specific weight than aluminum and iron but has low formability and a large springback. For this reason, the forming of the magnesium alloy is mainly performed in warm temperatures.

Elevated temperatures improve the ductility and formability of AZ31B. Simultaneously, the forming forces and springback occurrence decrease [2]. During the V-bending process using AZ31B, as the forming temperature increases and the punch radius decreases, the springback decreases. This is because the springback is affected by the elastic modulus and yield stress of the material. The modulus of elasticity does not change significantly with a temperature change, whereas the yield stress of AZ31B sheet decreases with increasing temperature. Therefore, the springback decreases as the forming temperature increases [3]. Additionally, deep drawing with magnesium alloy increases the LDR (limit drawing ratio) value as the forming temperature increases. But after 300 °C, the LDR value decreases due to the reduction of the local cross-section of the material [4].

However, as the forming temperature increases, the magnesium alloy exhibits different behavior such as creep and stress relaxation. Creep behavior is divided into three stages. The first one is a primary creep stage in which the creep strain rate gradually decreases. The second is a secondary creep stage in which the creep strain rate is constant. Lastly, a tertiary creep stage in which the rupture occurs as the creep strain rate increases rapidly. Within the temperature range from 423 to 443 K, the creep deformation rate increases significantly with increasing stress and temperature [5]. Additionally, at a forming temperature of 423 to 443 K, the creep behavior of magnesium alloy sheet increases the creep deformation and creep strain rate with increased forming temperature and a higher level of stress [6].

The creep behavior of AZ31B influences the residual stress formation and springback occurrence. When three-point bending was performed using a magnesium alloy sheet at room temperature, the specimen held in a trough after forming showed a tendency to decrease in springback over time. These results indicate that creep and creep recovery occurred [7]. Similarly, in the L and V bending processes, springback is reduced as the increasing holding time of the material in the die increases. In particular, the higher the forming temperature, the greater the decrease in springback as the increasing holding time of the material in the die [8]. These time-dependent characteristics of the material appear not only in AZ31b but also in other materials—for example, AC170PX aluminum [9], AHSSs, and TRIP [10,11]. It has been claimed that materials with higher yield strength showed more significant variation in the nearly saturated time-dependent springback angle, approximately proportional to the time-independent springback angle.

As in the above studies, there are other methods to get rid of the springback by removing residual stress, which does not use the time-dependent characteristics. For instance, in order to remove residual stress in the bending process using AZ31b, there is a method of reducing springback using electricity [12]. The reason for the decrease in springback is that the electric pulse can induce detwinning, which contributes to stress relaxation [13].

At present, much research has been conducted on the formability, creep, and stress relaxation characteristics of magnesium alloy. However, few studies have been conducted on the relationship between creep and stress relaxation characteristics and plastic deformation on the proper sheet metal forming condition (250 °C).

In this study, the time-dependent springback of AZ31B at warm conditions was studied. Tensile tests were conducted to analyze the mechanical properties of AZ31B at different temperatures. In addition, creep and stress relaxation tests were conducted to analyze the creep characteristics of AZ31B according to the temperature and pre-strain. To model the creep deformation, the stress exponent of creep strain was measured. This time-dependent behavior characteristic of magnesium alloys also affects the manufacturing process, especially on springback. We will present some examples of the time-dependent springback of AZ31B in the V- and L-bending process at various forming temperatures and conditions.

## 2. Materials and Methods

### 2.1. Uniaxial Tensile Test

**Figure 1:**
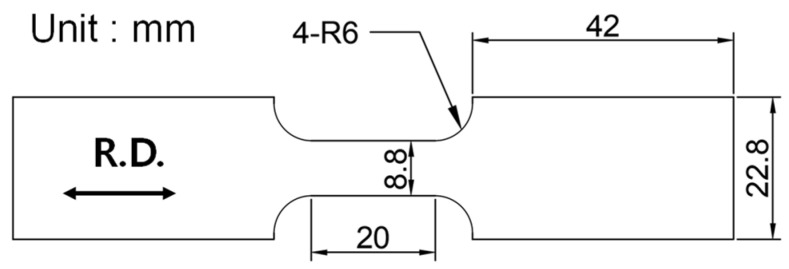
The schematic of the tensile test specimen.

Tensile tests were conducted to analyze the mechanical properties of AZ31B according to the forming temperature. The chemical composition table of the magnesium alloy is shown in Table 1. The specimens were cut in the rolling direction (R.D.) and the gauge was 20 mm long, 8.8 mm wide, and 1 mm thick [14]. The schematic diagram of the tensile test specimen is shown in Figure 1.

**Figure 2:**
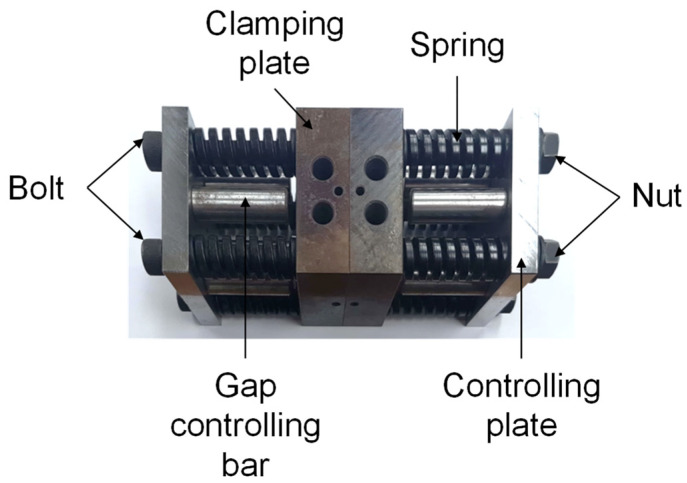
Pre-drilled clamp set for controlling the temperature of the material.

**Figure 3:**
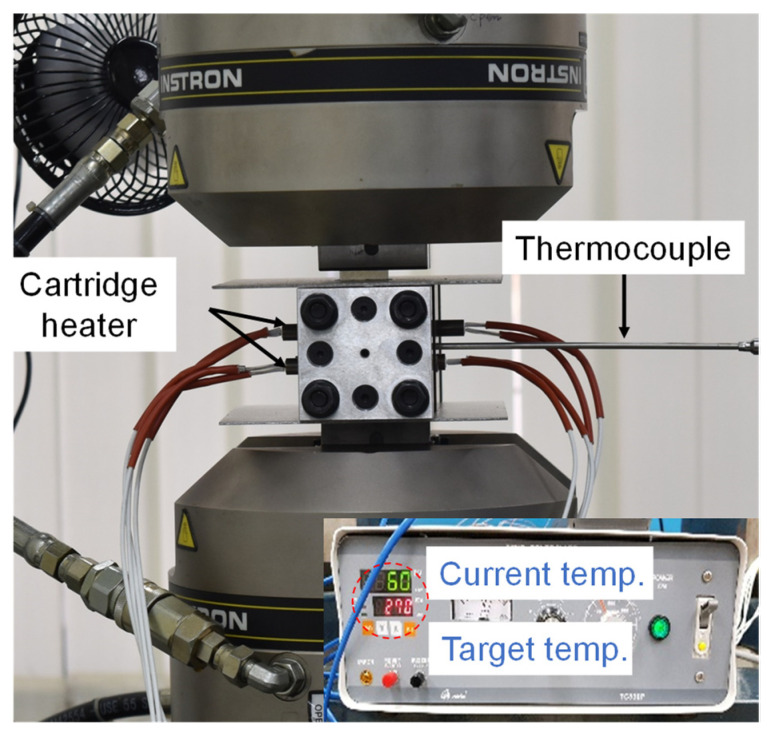
Experimental setup for tensile, creep, and stress relaxation tests.

In order to achieve the target forming temperature of the material, an experimental device as shown in Figure 2 was employed. The temperature of the material was controlled with a heating device. A circular hole was machined into the clamping plate to insert the cartridge heater. Compression coil springs, bolts, and nuts were used to allow the clamping plate to contact the clamping plate and the specimen. The material’s temperature was measured by attaching a K-type thermocouple to increase the reliability of the target temperature. The experiment was carried out after the temperature of the material reached the target temperature. The speed of the crosshead during the test was 12 mm/min, and the target temperature of the material was set to room temperature, 50 °C, 100 °C, 150 °C, 200 °C, and 250 °C. The equipment used in the experiment is shown in Figure 3.

### 2.2. Creep and Stress Relaxation Experiment

Creep and stress relaxation experiments were conducted to analyze the time-dependent mechanical behavior of AZ31B. The schematics of the test specimen and the device shown in Figure 3 are the same as the tensile test. The creep and stress relaxation behavior of magnesium alloy sheets were analyzed by applying a constant displacement and a constant load after tension to the target pre-strain. The time of maintaining constant load and displacement for each experiment is 0~1000 s. The magnitude of the pre-strain was the value of the strain at 50%, 75%, 90%, and 100% of the ultimate tensile strength. The pre-strain with total elongation of 40% was added in the experiment. The corresponding value of the pre-strain at 250 °C is shown in Table 2. For example, *ε*_50_ means that the pre-strain was 0.007, which is the strain at 50% of the ultimate tensile strength. These strains were set as the reference strains because the deformation at 250 °C focuses more on this work.

The speed of the crosshead during the test was 12 mm/min (strain rate = 0.01/s). In the case of the creep test, the force of UTM was controlled constantly. In the case of a stress relaxation test, the displacement of the crosshead was controlled to measure the decrease of the force.

### 2.3. L-Bending and V-Bending Experiments

**Figure 4:**
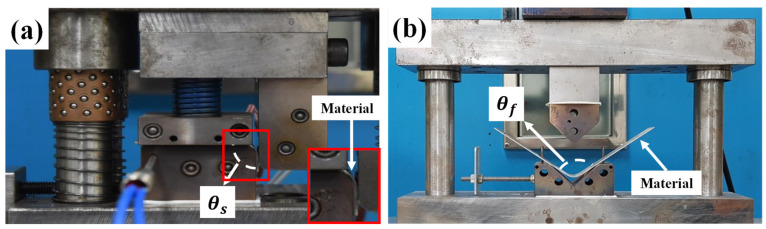
The die set of (**a**) L-bending and (**b**) V-bending [15].

In order to investigate time-dependent springback, the V- and L-bending processes were employed. The die sets for the V- and L-bending process presented in Figure 4 were employed [15]. The length of the specimen was 60 mm and 200 mm in the L- and V-bending, respectively. The width of the specimen was 30 mm, and the thickness of the material was 1 mm in both experiments. The speed of the crosshead and the punch was 150 mm/min. The forming temperature is 250 °C. The springback (Δ*θ* = *θ_f_* − *θ_s_*) of each process was the difference between the angle of the product after the holding time of the material in die at the bottom dead center (*θ_f_*) and the angle of the material at the bottom dead center after forming (*θ_s_*), where the bottom dead center means that the punch in the die has descended to the target stroke. The springback of the material was measured according to the holding time in the die set.

Unlike previous experiments, we conducted bending experiments with the different punch and die radius values. Additionally, microscopic investigations were conducted. Each bending experiment was conducted with the different die radius in the L-bending process and the different punch radius in the V-bending process. The corner radius of the L-bending die is 4 mm and 6 mm, and the radius of the V-bending punch is 1 mm and 6 mm.

### 2.4. Microstructure Observation

**Figure 5:**
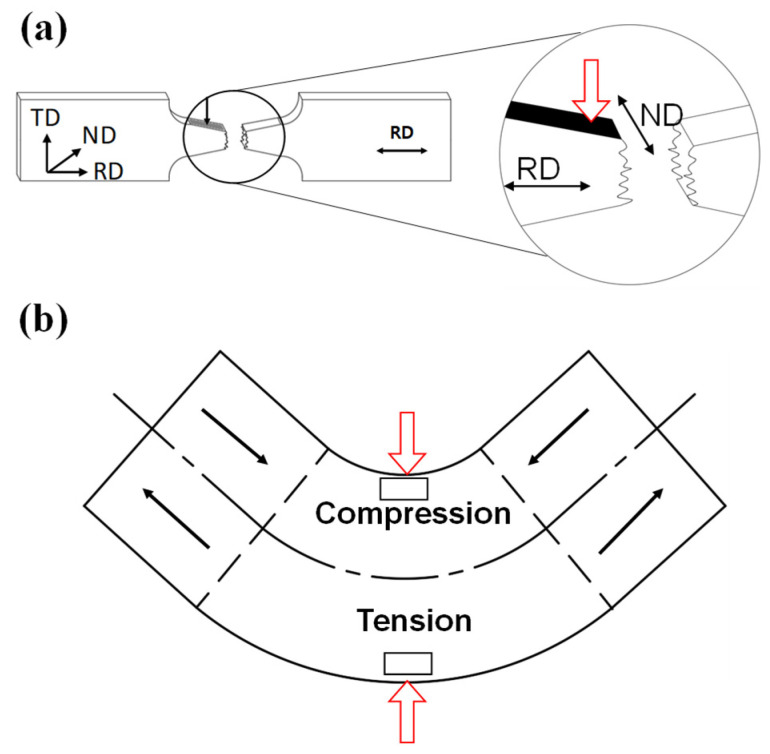
Microstructure observation region in (**a**) the stress relaxation specimen and (**b**) the bending specimen.

Microstructure changes concerning the strain and time of AZ31B were analyzed during warm forming. The cold mounting was performed after cutting the deformed specimen. Mounted samples were polished using with SiC papers and alumina powders. After that, in order to observe the microstructures, etching was performed using a corrosion solution (Acetic acid 75 mL, ethanol 70 mL, distilled water 10 mL, and picric acid 4.2 g) (SAMCHUN Chemicals, Seoul, Korea). Finally, the etched specimens were examined for microstructure through an optical microscope (Motic, Vancouver, BC, Canada). The observed plane of the specimen includes the normal direction (ND) and the rolling direction (RD) as illustrated in Figure 5. In V-bending specimens, the microstructure was observed microstructures in the inner and outer regions to compare compressive and tensile behavior. The grain size was measured using the line-intercept method.

## 3. Results and Discussion

### 3.1. Tensile and Creep Experiment

#### 3.1.1. Uniaxial Tensile Test Results with Respect to Forming Temperature

**Figure 6:**
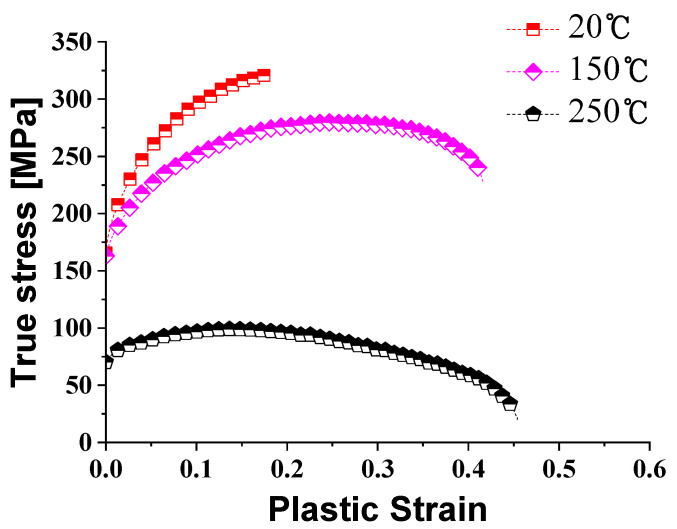
True stress-strain curve of AZ31B according to the temperature.

Through tensile tests, the mechanical properties of AZ31B were analyzed with respect to temperatures. The true stress-strain curves are shown in Figure 6. The ultimate tensile strength at room temperature was 321.57 MPa, and the ultimate tensile strength at 250 °C was 118.94 MPa, which was an about 63.01% decrease compared to room temperature. As the forming temperature increases, the total elongation increases, and the flow stress decreases. This result is the same as the previous study. Work softening due to recrystallization proceeds rapidly after 150 °C, which is the recrystallization temperature of magnesium [16,17].

**Figure 7:**
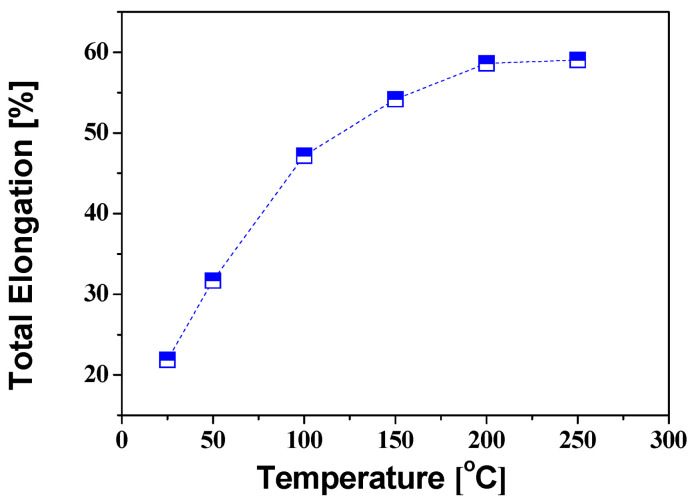
Relationship between the total elongation and the temperature of AZ31B.

The total elongation of AZ31B with various temperatures is shown in Figure 7. As shown in Figure 7, the total elongation is 59.02% at 250 °C, which is about three times higher than the 21.86% elongation at room temperature. The increase is due to the activation of the non-basal slip surface in the HCP structure of the magnesium alloy during warm forming [18]. As a result, as the forming temperature increases, the total elongation increases and the rate of increase in elongation decreases and converges to 60% after 200 °C. For the compressive behavior of magnesium alloys at room temperature, the tensile yield stress is about twice that of the compressive yield stress, and this difference decreases with increasing forming temperature [19].

**Figure 8:**
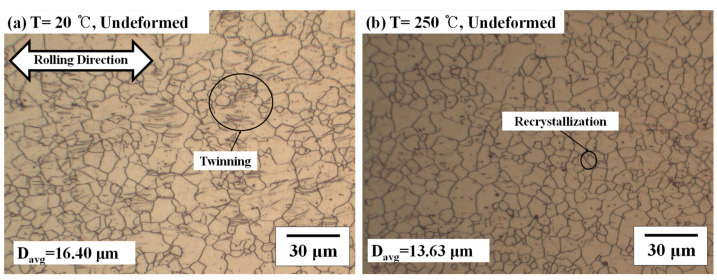
The initial microstructure of AZ31B at (**a**) 20 °C and (**b**) 250 °C.

The microstructure was analyzed to study the behavior characteristics according to temperatures. The microstructures of the initial AZ31B at 20 °C and 250 °C are shown in Figure 8. At 20 °C, twinning, which results from the rolling process, is observed in the initial microstructure of AZ31B, as shown in Figure 8a. The deformation is limited due to twinning and less slip planes.

**Figure 9:**
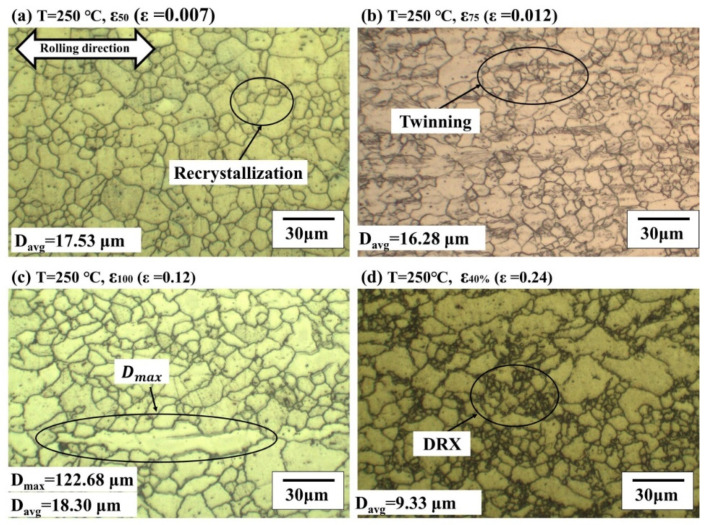
The microstructure of AZ31B after tensile tests at 250 °C with different pre-strains of (**a**) *ε*_50_ (*ε* = 0.007), (**b**) *ε*_75_ (*ε* = 0.012), (**c**) *ε*_100_ (*ε* = 0.12), and (**d**) *ε*_40%_ (*ε* = 0.24).

On the other hand, at 250 °C, the initial microstructure recrystallized as the temperature rose above the recrystallization temperature (150 °C) of the magnesium alloy as shown in Figure 8b. Due to recrystallization, the average grain size was 16.40 μm at 20 °C, and the average grain size (D_avg_) was 13.77 μm at 250 °C, which is 2.77 μm smaller. As the temperature increases, twinning caused by compressive stress at room temperature disappears due to thermal annealing. At 250 °C, the microstructure changes according to the strain (*ε*) is shown in Figure 9. The experiment was stopped immediately after reaching the target strain. Since the tensile deformation occurred above the recrystallization temperature (150 °C) of AZ31B, recrystallization occurs at all strains. Except for Figure 9d, the average grain size showed a maximum difference of 2.02 μm. After passing the ultimate tensile strength, the load decreases. At the same time, a lot of dynamic recrystallization (DRX) occurred suddenly, as shown in Figure 9d [20]. As a result, after uniform elongation, dynamic recrystallization occurs rapidly, and results in work softening [21]. In addition, due to dynamic recrystallization, the average grain size is about 0.5 times compared to uniform elongation.

#### 3.1.2. The Creep Behavior with Various Pre-Strain and Forming Temperature

**Figure 10:**
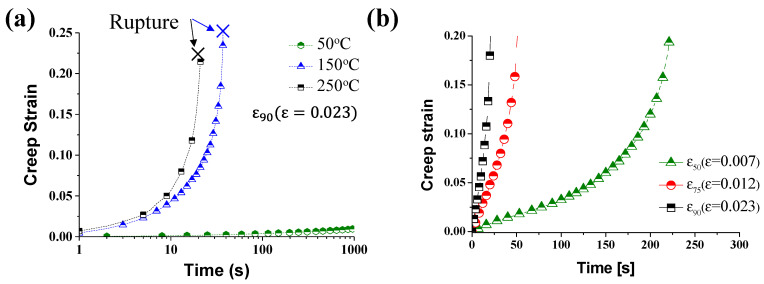
The creep strain–time graph according to (**a**) different temperatures at *ε*_90_ (*ε* = 0.023) and (**b**) different pre-strain at 250 °C.

In this study, we analyzed the creep behavior of AZ31B according to the applied pre-strain in warm forming. After tensile tests, the force applied to the material remained constant, and the displacement was measured. The characteristics of the creep behavior according to forming temperatures and pre-strains are shown in Figure 10. The rupture times according to temperatures and pre-strains are presented in Table 3. Figure 10a shows creep behavior with forming temperature at the same pre-strain of *ε*_75_.

Magnesium alloy sheet enters the secondary creep section within 30 s at 150 °C and 250 °C. Rupture did not occur at 20 °C and 50 °C. The creep rupture time at 150 °C was about 38 s, and at 250 °C was 21 s with the pre-strain of 0.023 (*ε*_90_). Creep rupture time has been significantly shortened as the temperature increases. Figure 10b shows the creep behavior according to the pre-strain at the same forming temperature. As the pre-strain increased, the creep rupture time decreased by 205 s from 256 s at *ε*_50_ (*ε* = 0.007) to 21 s at *ε*_90_ (*ε* = 0.023). Large deformation of the magnesium alloy and the high forming temperature result in weakening of the creep resistance.

**Figure 11:**
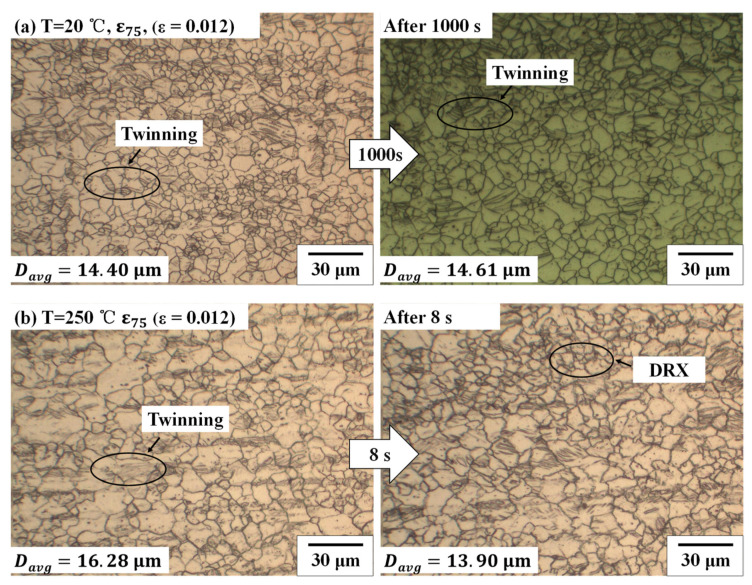
Change of the microstructure after the creep test with the pre-strain of *ε*_75_ (*ε* = 0.012) (**a**) at 20 °C with the holding time of 1000 s and (**b**) at 250 °C with the holding time of 8 s.

The microstructure changes in the creep test at 20 °C and 250 °C with the pre-strain of *ε*_75_ (*ε* = 0.012) are shown in Figure 11. We have compared the microstructural change after a holding time of 1000 s (secondary creep stage) after the tensile test with the pre-strain of *ε*_75_ (*ε* = 0.012) at 20 °C, as presented in Figure 11a. Figure 11b presents the microstructure change at 250 °C with the holding time of 8 s (secondary creep stage). At 20 °C, the change of the microstructure is not significant. The average size of the microstructure increased by 1.46%. On the other hand, at 250 °C, after 8 s, the average size of the microstructure decreased 14.62% due to dynamic recrystallization.

In the creep test at 20 °C, the rupture did not appear until the constant load holding time was 1000 s, while the rupture occurred at the constant load holding time of 21 s at 250 °C. The mechanism of the creep at high temperatures is as follows; a significant amount of dislocation slips is activated on basal and non-basal planes during the primary creep stage; then, dynamic recrystallization (DRX) occurred; finally, the initiation of the cracks on the grain boundaries expanded [22,23,24].

#### 3.1.3. The Stress Relaxation Behavior with Various Pre-Strains and Forming Temperatures

**Figure 12:**
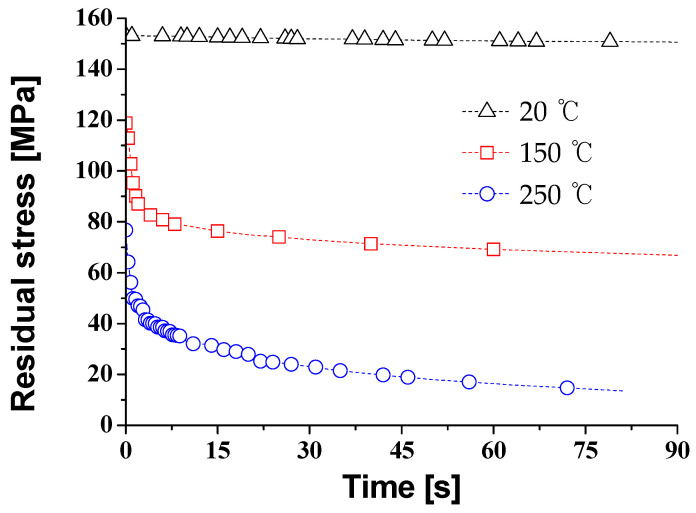
The residual stress according to the holding time at the pre-strain of *ε*_75_ (*ε* = 0.012) with different temperatures.

In order to study the stress relaxation behavior, the displacements of the tensile grip after the tensile test remained constant. At the same time, the decrease of the load was measured. The load was divided by the initial area (engineering stress); the engineering stress was measured for the residual stress. Figure 12 presents the distribution of the residual stress with respect to the temperature at the pre-strain of *ε*_75_ (*ε* = 0.012). The stress relaxation time was the time when the stress decreased to 37% of the initial load. The amount of stress relaxation occurred between the stress at 0 s and the stress at 30 s. The stress relaxation time and the amount of stress relaxation at each forming temperature are shown in Table 4.

At 20 °C, the residual stress was 153.3 MPa after the tensile test, and the load was not reduced much as the time increased. On the other hand, in the case of 250 °C, the stress after the tensile test was 76.75 MPa, and the stress was 22.85 MPa after 30 s of the holding time. The stress relaxation time is 150 s at the forming temperature of 150 °C, and the stress relaxation time is 19 s at 250 °C. These results show that as the forming temperature increases, the reduction of stress increases, and the stress relaxation time was reached quickly.

**Figure 13:**
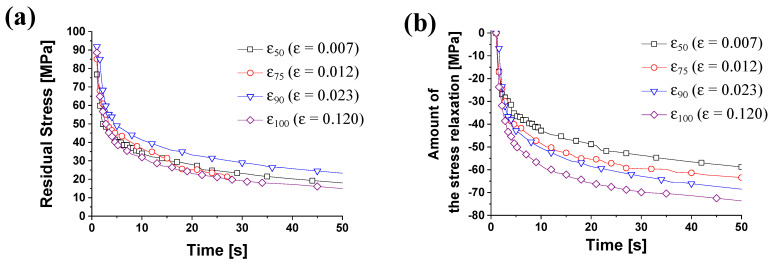
The stress relaxation at 250 °C with different pre-strain values; (**a**) residual stress and (**b**) amount of the stress relaxation.

The distribution of the residual stress according to the pre-strains at 250 °C was compared in Figure 13a. As the holding time after tensile tests increases, the stress of the material decreases exponentially. At all pre-strains, the stress tended to converge from 30 s at constant holding time. In order to compare the reduced stress amount, the decreased forces were compared in Figure 13b. As the initial stresses are different, we calculated the amount of the stress reduction, which started from zero. The stress relaxation time was defined as the same method.

The stress reduction amount is 53.3 MPa at *ε*_50_ and 69.9 MPa at *ε*_100_ for 30 s of constant displacement holding time. Under the same holding time, the load reduction occurs more when the plastic deformation is large than when it is small. The stress relaxation time was 46 s at the strain *ε*_50_ and 10 s at *ε*_100_. As the pre-strain increased, the stress relaxation time tended to decrease. Table 5 shows the load reduction and stress relaxation times for each pre-strain. It means that large plastic deformation results in the large reduction of the load.

**Figure 14:**
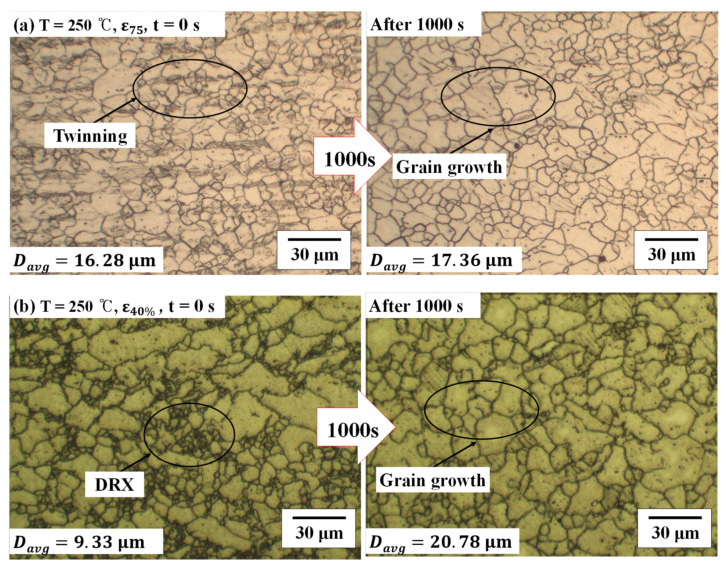
Change of the microstructure after the stress relaxation test (**a**) at the strain of *ε*_75_ (*ε* = 0.012) with the holding time of 1000 s, (**b**) at the strain of *ε*_40%_ (*ε* = 0.24) with the holding time of 1000 s.

The effects of the stress relaxation in the microstructure changes were compared according to the holding time at 250 °C. The microstructure at 20 °C was not shown because the change of the microstructure is not significant. When the pre-strain was 0.012 (*ε*_75_), the microstructure changes were presented in Figure 14a,b. The grain growth was 1.08 μm at the pre-strain of 0.012 and 11.45 μm at the pre-strain of 0.24. Additionally, the residual stress, which is caused by plastic deformation of the material, decreased through the recovery and recrystallization process that appeared during the warm forming. This is because the twinning, which occurred during tensile deformation, disappears as the constant displacement holding time increases. As a result of creep and stress relaxation, the magnesium alloy has high resistance to creep and load reduction due to the low number of slip surfaces and high critical resolved slip stress at room temperature. On the other hand, as the forming temperature increases, the load required for deformation decreases due to the increase of slip surface and low critical resolved slip stress, thereby lowering the resistance to creep and load reduction [25].

#### 3.1.4. Stress Exponent of Creep Strain

The stress exponent for the creep strain at the forming condition of AZ31B was calculated. The creep behavior characteristics of the magnesium alloy according to the forming temperature and the constant load holding time were analyzed. From these results, by the following method, the creep strain was represented by the simple power law formula below:

(1) $$\varepsilon_{c}=A\sigma^{n}t^{m}\;$$

where *A*, *n*, *m* are the temperature-dependent parameters. The primary creep stage of AZ31B is very short compared to the secondary creep stage, as shown in Figure 10. Consequently, the primary creep stage is not considered in the modeling. The experimental formula expressing the creep strain rate as a function of stress and temperature was expressed in Equation (2). Equation (2) shows the dependence of the normal creep rate (secondary creep) on the stress. To find the parameters A and *n*, take the logarithm in Equation (2) and make it into a linear equation (Equation (3)).

(2) $$\dot{\varepsilon_{c}}=A\sigma^{n}\;$$

(3) $$ln\left(\dot{\varepsilon_{c}}\right)=lnA+nln\sigma \;$$

The parameters *A* and *n* were obtained through Equation (3). For the stress exponent 250 °C, *n* is 4.80 and the value of the parameter *A* is 4.46 × 10^−12^. The stress exponent obtained in this study was compared with the previous results [26,27].

**Figure 15:**
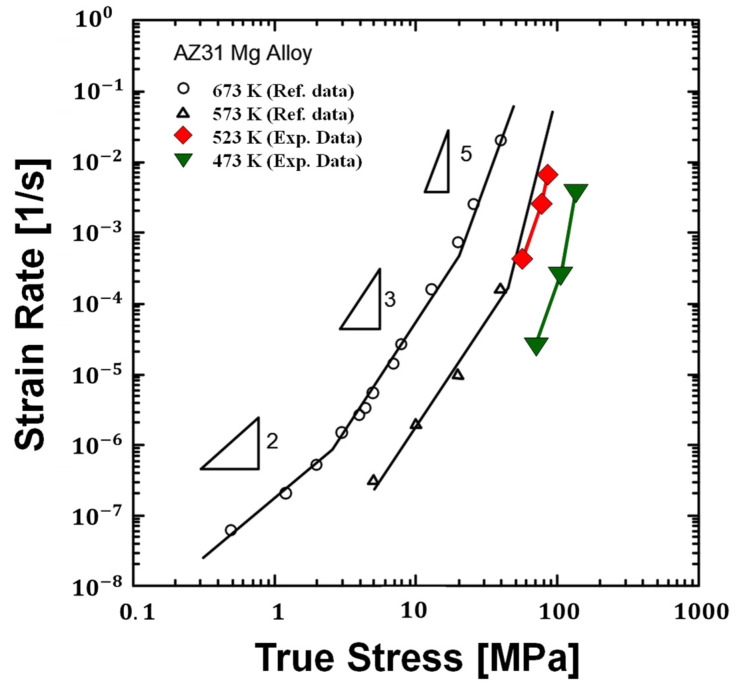
Steady state strain rate against stress for specimens. The reference data was obtained from Chung and Kim [26].

Figure 15 represents the creep rate for the secondary creep strain corresponding to each temperature. Our results present the stress exponent at the proper forming conditions of AZ31B, such as the forming temperature and stress values. Using these creep properties, the time-dependent mechanical behavior can be predicted. Finite element analysis with the time-dependent mechanical behavior is required to design the manufacturing process of AZ31B at warm conditions, and related research is underway.

#### 3.1.5. Validation of Creep Parameters Using Finite Element Simulation

The commercial FE code ABAQUS v6.14 was employed to simulate the magnesium alloy sheets’ creep behavior. The material behavior was assumed to be isotropic at a forming temperature of 250 °C. The creep model of the magnesium alloy sheet used a simple power law. The model parameters were obtained through a high-temperature tensile test, and the process is as described in Section 3.1.4. Table 6 shows the properties of the magnesium alloy used in the simulation.

The geometry of the tensile specimen was the same as that of experiments shown in Figure 1. Only one-quarter of the model was meshed due to symmetry. We conducted simulation in the 2D plane stress condition. The model contains four-node 880 elements (CPS4R).

**Figure 16:**
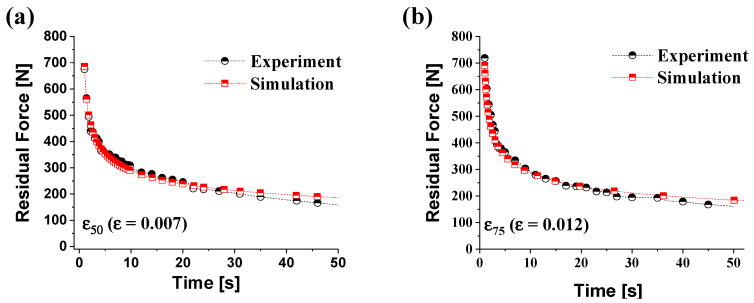
The comparison of the residual stress with the experimental and simulation results at the forming temperature of 250 °C and pre-strain of (**a**) *ε*_50_ (*ε* = 0.007), (**b**) *ε*_75_ (*ε* = 0.012).

In order to verify the creep parameters obtained through the experiment, the stress relaxation test results and the simulation results were compared. Figure 16 shows the experimental results and simulation results under the conditions of the forming temperature 250 °C and the pre-strain *ε*_50_ (*ε* = 0.007, Figure 16a), *ε*_75_ (*ε* = 0.012, Figure 16b). Both the experimental and simulation results showed a rapid decrease of the forming load until 30 s after the pre-strain and showed a tendency to converge. The amount of residual force reduction on the experiment and simulation during the constant displacement holding time of 30 s is presented in Table 7. The error based on the experimental results of pre-strain *ε*_50_ and *ε*_75_ is 1.08% and 2.74% after pre-strain, respectively. The errors are 3.89% and 7.53%, respectively, after 30 s of constant displacement holding time. Simulation results showed good agreement with the experiments. It was found that the creep parameters obtained from the experiments were valid.

### 3.2. Time-Dependent Springback in Bending Deformation

#### 3.2.1. Time-Dependent Springback with Different Temperature

**Figure 17:**
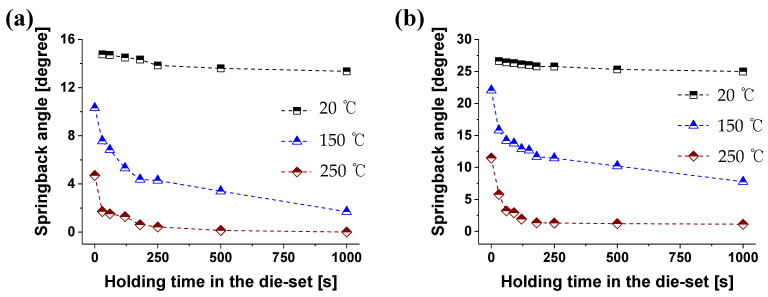
The springback according to holding time in the die set of (**a**) L-bending and (**b**) V-bending with different forming temperatures.

The characteristics of time-dependent springback behavior were analyzed through the V- and L-bending processes with the die sets shown in Figure 4. The die radius of the L-bending process is 6 mm, and the punch radius of the V-bending process is 6 mm. The springback behavior according to the temperature during forming and the holding time in the die set is shown in Figure 17.

The initial angle (*θ_s_*) of the material at the bottom dead center during the bending process was 90.39° for the L-bending and 90.5° for the V-bending. In both bending processes, the springback decreased as the forming temperature increased; these results are consistent with the results of previous studies. In room temperature, the springback change with respect to the holding time in the die set is not significant. Above 150 °C, the time-dependent springback characteristics occurred. In the L-bending process, when the holding time of the material in the die at room temperature was maintained for 1000 s, it was reduced by 1.41° compared to 0 s. On the other hand, it decreased by 8.61° at 150 °C.

The relationship between the holding time in the die set and springback angle shows similar trends with the relationship between residual forces and holding time. In the creep and stress relaxation characteristics of the magnesium alloy sheet, as the forming temperature and the pre-strain increased, the secondary creep section entry and stress relaxation occurred faster. This relationship also applies to the time-dependent springback characteristics.

#### 3.2.2. Time-Dependent Springback with Different Stress Levels

**Figure 18:**
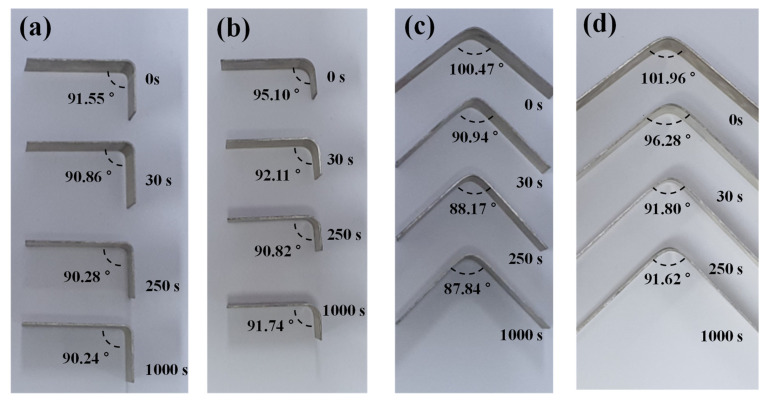
Deformed geometry after springback at 250 °C with various holding times in the die set; (**a**) L-bending with the die radius of 4 mm; (**b**) L-bending with the die radius of 6 mm; (**c**) V-bending with the punch radius of 1 mm; (**d**) V-bending with the punch radius of 6 mm.

We conducted more bending tests with different punch radii. For the V-bending process, experiments with punch radii of 1 mm and 6 mm were conducted. For the L-bending process, experiments with die radii of 4 mm and 6 mm were conducted. For the V-bending and L-bending, lower radius values of the punch and the die mean concentrated deformation. It resulted in a high equivalent plastic strain. The time-dependent springback characteristics with different conditions are shown in Figure 18.

**Figure 19:**
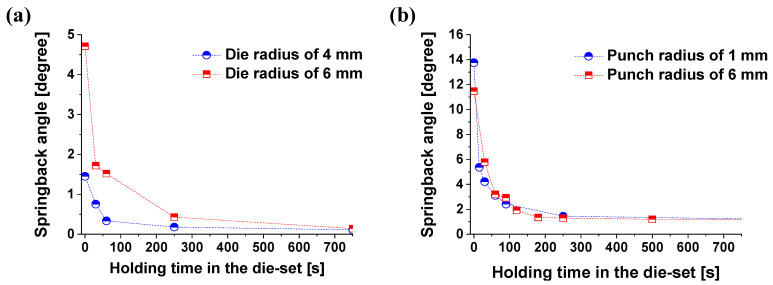
The springback graph of AZ31B according to punch radius and time on 250 °C during the (**a**) L-bending and (**b**) V-bending processes.

In order to compare the springback reduction, the springback angle change according to time was compared for each case, as shown in Figure 19. In the bending process, the smaller the punch radius and die radius, the higher the stress of the material. Therefore, in the die radius of 4 mm (L-bending) and the punch of 1 mm (V-bending), the creep stress is high, and a decreasing rate also occurs rapidly. This behavior is the same as that of the previous section. In both the L- and V-bending processes, the springback tended to converge from 250 s. In the case of the L-bending process, the springback change with the holding time of 60 s was reduced by 76.89% when the die radius was 4 mm and 67.73% when the die radius was 6 mm. In the case of the V-bending process, the springback change in the holding time of the material in the die from 0 s to 60 s decreased by 77.38% when the punch radius was 1 mm and 74.52% when the punch radius was 6 mm. Figure 19 presents the deformed geometry to punch radius and holding time in the die set at 250 °C after the L- and V-bending processes.

#### 3.2.3. FE Modeling

**Figure 20:**
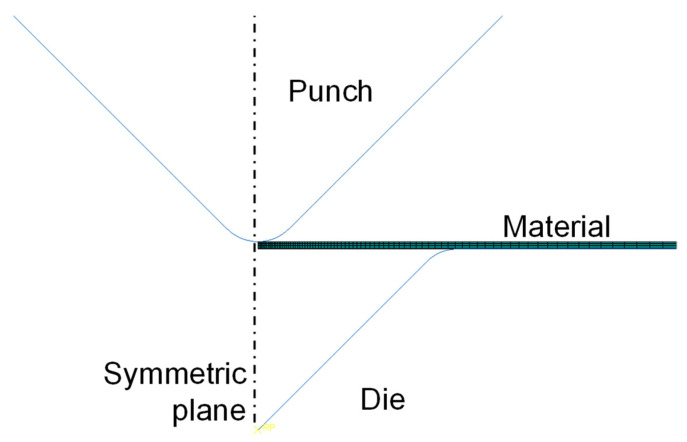
2D symmetric finite element model for the V-bending process.

The V-bending process was simulated via finite element analysis using a 2D symmetric model, as shown in Figure 20. The punch and die were modeled using an analytically rigid surface. The specimen was meshed using 2D plane strain, quadrilateral, and CPE4R elements, and the number of elements in the specimen was 700. The creep parameters’ power-law multiplier (A) and stress exponent (*n*) are 4.46 × 10^−12^, and 4.80, respectively, and the elastic modulus, Poisson ratio and yield stress are 10.40 GPa, 0.30, and 78.63 MPa, respectively. The displacement of the punch was held during the holding time in the die set.

#### 3.2.4. Simulation Results of the Bending Process

**Figure 21:**
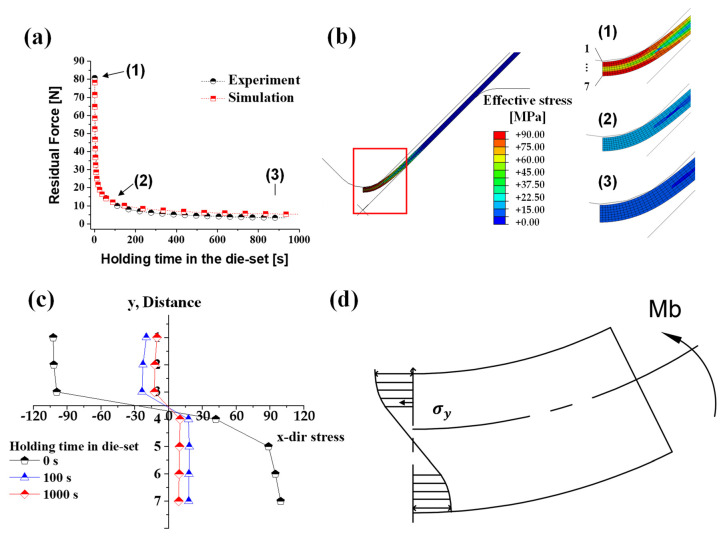
(**a**) The comparison between experiment and simulation results of a residual force; (**b**) visualization of (**c**) stress distribution according to the holding time in the die set; (**d**) the schematic of stress distribution.

Figure 21a shows the residual force of experiments and simulations with respect to the holding time in the die set at a forming temperature of 250 °C. Identical to the stress relaxation test, the residual load tends to be relieved in a short time. As mentioned in the microstructure section above, stresses of material from the forming process decrease in a short time due to rapid dynamic recrystallization. This phenomenon is also significantly related to the time-dependent springback. When the punch is fixed after V-bending, elastic recovery and recrystallization of the material occur within a short time. As the holding time in the die set increases, the residual force and stress from the deformation decrease.

Figure 21b,c presents the stress distribution along the thickness direction from the bending process to the material holding time of 1000 s. After the bending process, stress decreased rapidly from the material holding time of 100 s in the die set, and then the stress was relieved slowly. Additionally, the further away from the neutral axis (closer to the surface of the material), the faster the rate of stress relaxation. Consequently, as the holding time in the die set increases, the springback of the material decreases.

#### 3.2.5. Microstructure Change in the Bending Deformation with Respect to Time

**Figure 22:**
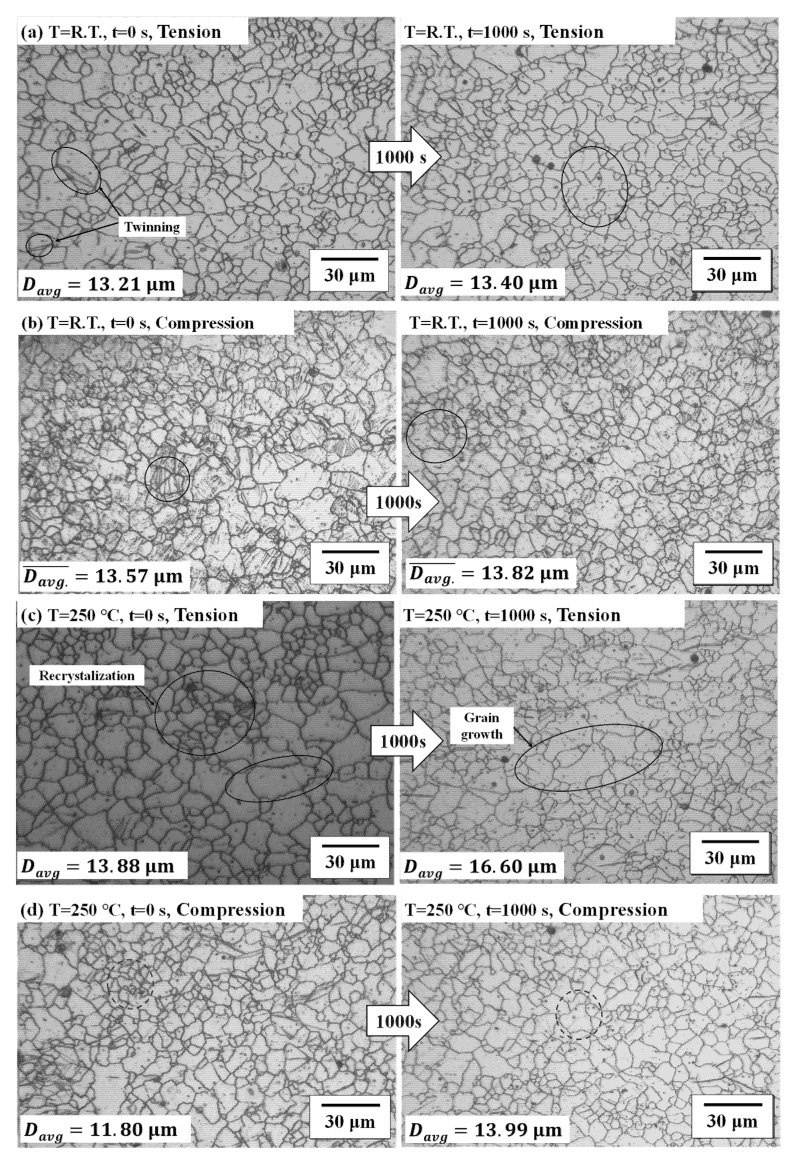
The microstructure of AZ31B after V-bending process according to material holding time (**a**) on the outer side (tensile deformation) at 1000 s and 20 °C, (**b**) on the inner side (compressive deformation) at 1000 s and 20 °C, (**c**) on the outer side at 1000 s and 250 °C, and (**d**) on the inner side (compressive deformation) at 1000 s and 250 °C.

We have compared the microstructural change in the bending deformation with holding time and deformation modes. Figure 22 shows the microstructure change result according to the increase in the material holding time in the die when the V-bending process was at room temperature and when it was at 250 °C with a punch radius of 6 mm.

The four figures on the left of Figure 22 show that twinning occurred in the compressive zone (inner side of the bent product) more than the tensile zone. The tensile behavior of magnesium alloy is dominated by slip. On the contrary, the compression behavior is dominated by twinning. This causes asymmetry of the magnesium alloy sheet’s tensile compression behavior [28].

At 20 °C, the shape of the grains shown in Figure 22a,b did not change significantly, as the holding time in the die increased. The average grain size also did not change considerably, with a difference of 0.19 and 0.25 μm, respectively. On the other hand, the microstructures changed with an increase in holding time in the die at 250 °C, as shown in Figure 22c. The average grain size shows a difference of 3.47 μm. Figure 22d presents the microstructure change on the inner side, which shows compression deformation. In the compressive zone at 250 °C, the average size of the microstructure increases.

The average size of the grain increased with the holding time in the die set at both sides (tensile zone and compressive zone). These results show a similar tendency to microstructural changes in stress relaxation according to the forming temperature in the previous section.

In both the L- and V-bending processes, the springback decreased as the holding time of the material in the die increased. This is because the internal stress decreases as the material remains in the die, reducing the springback. In addition, new crystals are generated in the magnesium alloy sheet at 250 °C, and dynamic recrystallization occurs as a result of deformation. The material is deformed and maintained in the die, newly generated crystal grains undergo growth, and internal energy decreases, thereby reducing springback. These results are similar to those of the microstructures that change during stress relaxation according to the magnesium alloy’s forming temperature.

In summary, the springback decreased along the holding time due to the internal stress relaxation inside the material. It was found that these results have a similar tendency towards the stress relaxation behavior according to the pre-strain in the magnesium alloy sheet. However, the springback reduction in the L-bending process according to the plastic deformation amount shows a slow decrease rate compared to the V-bending process. The reason for this is as follows: in the case of V-bending, the forming position is symmetrical with respect to the center; however, L-bending is asymmetric. It makes forming position of the L-bending move in the bending zone. Therefore, the forming part of the material located in the bending zone after forming is in a state of relatively low stress [29]. In Section 3.1.3, it was mentioned that the lower the pre-strain—that is, the lower the stress level—the slower the residual stress decreases. Therefore, it can be seen that the springback decreases more slowly in L-bending than in V-bending.

Based on the above results, the holding time of the material in the die significantly affects the reduction of the springback. In particular, it was shown that the springback was reduced more than 70% until the holding time in the die set of 60 s. Therefore, even in the actual forming process, the process must be designed in consideration of the holding time in the die set.

## 4. Conclusions

In this study, the characteristics of springback behavior were analyzed by applying creep and stress relaxation behavior of the pre-strain of a magnesium alloy sheet to a V-bending test.

1. The magnesium alloy sheet has a lower resistance to creep and stress relaxation as the forming temperature increases. It is confirmed that the resistance is lowered due to static and dynamic recrystallization due to temperature and deformation.
2. In the creep test, the time to rupture was shortened to 339 s at 100 °C and 21 s at 250 °C at pre-strain of *ε*_90_. At the same forming temperature of 250 °C, the time to rupture was shortened to 226 s at *ε*_50_ and 21 s at *ε*_90_.
3. In the stress relaxation test, at the same pre-strain *ε*_75_, the load reduction was 5% at room temperature and 73% at 250 °C. In addition, the load relaxation time was 900 s at 150 °C and 19 s at 250 °C. The higher the forming temperature, the shorter the load relaxation time. It can be seen that as the forming temperature increases, the internal stress decreases due to recrystallization and the rate of stress released is also fast.
4. In the stress relaxation test at the same pre-strain (*ε*_75_), the residual force at constant holding time 0 s and 250 °C is 76.7 MPa and the residual force is 20.7 MPa after a holding time 1000 s at 250 °C. It was reduced by 73% based on the initial load. On the other hand, at the same pre-strain and at room temperature, the residual force at a constant holding time of 0 s is 152.3 MPa and the residual force is 144.9 MPa after a holding time of 1000 s at room temperature. It was reduced by 5% based on the initial load. In addition, under the same forming temperature (250 °C) condition, the initial load was reduced by 69% and 74% at pre-strain *ε*_50_ and *ε*_90_, respectively. As the pre-strain increases, the rate of stress relaxation is faster.
5. In the warm V-bending, the springback reduction according to the forming temperature was reduced by 59% from 28.11° at room temperature to 11.46° at 250 °C. Springback reduction according to the holding time of the material in the die at 250 °C was 11.46° at 0 s, 1.12° at 1000 s, with a 90% reduction compared to the initial angle. This shows that the amount of springback decrease due to the increase in the holding time of the material in the die is greater than the amount of springback decrease due to the temperature of the material.

Through this, the formability of products using magnesium alloy sheets in the metal processing field is improved and the springback improves as expected.

## Figures and Tables

**Table 1:** The chemical composition of magnesium alloy AZ31B (wt.%).

Magnesium (Mg)	Aluminum (Al)	Zinc (Zn)	Manganese (Mn)	Silicon (Si)	Copper (Cu)	Calcium (Ca)	Iron (Fe)	Nickel (Ni)
97%	2.5–3.5%	0.6–1.4%	0.2%	0.1%	0.05%	0.04%	0.005%	0.005%

**Table 2:** The pre-strain value according to the forming load at 250 °C.

	50% of UTS (*ε*_50_)	75% of UTS (*ε*_75_)	90% of UTS (*ε*_90_)	100% of UTS (*ε*_100_)	e = 40% (*ε*_40%_)
Pre-strain	0.007	0.012	0.023	0.120	0.240
Applied load (N)	675.42	719.27	808.2	820.00	678.02

**Table 3:** The time of rupture according to the pre-strain and forming temperature.

The Time of Rupture (s)	20 °C	100 °C	150 °C	250 °C
*ε*_50_ (*ε* = 0.007)	- (Rupture did not occur)	-	-	226
*ε*_75_ (*ε* = 0.012)	-	-	269	54
*ε*_90_ (*ε* = 0.023)	-	339	38	21

**Table 4:** The amount of the stress relaxation and the stress relaxation time.

	Room Temperature	150 °C	250 °C
Stress relaxation time (s)	-	150	19
The amount of the stress relaxation after 30 s (MPa)	1.4	45.9	53.9

**Table 5:** The stress relaxation time and amount of stress relaxation according to the pre-strain.

Pre-Strain	0.007	0.012	0.023	0.120
Time of stress relaxation (s)	46	26	19	10
Amount of stress relaxation after 30 s (MPa)	53.3	59.6	62.8	69.9

**Table 6:** The properties of the magnesium alloy used to the simulation at a forming temperature of 250 °C.

Elastic Modulus © (GPa)	Poisson’s Ratio (υ)	Yield Stress (σ_y_) (MPa)	Power Law Multiplier (A)	Stress Exponent (*n*)
10.40	0.30	78.63	4.46 × 10^−12^	4.80

**Table 7:** The amount of residual force reduction in experiment and simulation at 250 °C.

Amount of Residual Force Reduction (N)				
Constant Displacement Holding Time	Pre-Strain of *ε*_50_ (*ε* = 0.007)		Pre-Strain of *ε*_75_ (*ε* = 0.012)	
	0 s	30 s	0 s	30 s
Experiment	675.42	205.90	719.27	195.27
Simulation	685.95	211.49	691.30	209.97
Error based on experimental results	1.08%	2.71%	3.89%	7.53%

## Data Availability

The data presented in this research study are available in this article.
